# Atypical Genotypes for Canine *Agouti Signaling Protein* Suggest Novel Chromosomal Rearrangement

**DOI:** 10.3390/genes11070739

**Published:** 2020-07-03

**Authors:** Dayna L. Dreger, Heidi Anderson, Jonas Donner, Jessica A. Clark, Arlene Dykstra, Angela M. Hughes, Kari J. Ekenstedt

**Affiliations:** 1 Department of Basic Medical Sciences, College of Veterinary Medicine, Purdue University, West Lafayette, IN 47906, USA; ddreger@purdue.edu (D.L.D.); clark542@purdue.edu (J.A.C.); 2 Wisdom Health, FI-00290 Helsinki, Finland; heidi.anderson@effem.com (H.A.); jonas.donner@effem.com (J.D.); 3 Coronado, Sturgeon County, AB T0A 1N4, Canada; arlenedykstra@gmail.com; 4 Wisdom Health, Vancouver, WA 98683, USA; angela.hughes@effem.com

**Keywords:** dog, coat color, colour, *ASIP*, duplication, gene, recombination, allele

## Abstract

Canine coat color is a readily observed phenotype of great interest to dog enthusiasts; it is also an excellent avenue to explore the mechanisms of genetics and inheritance. As such, multiple commercial testing laboratories include basic color alleles in their popular screening panels, allowing for the creation of genotyped datasets at a scale not before appreciated in canine genetic research. These vast datasets have revealed rare genotype anomalies that encourage further exploration of color and pattern inheritance. We previously reported the simultaneous presence of greater than two allele variants at the *Agouti Signaling Protein (ASIP)* locus in a commercial genotype cohort of 11,790 canids. Here we present additional data to characterize the occurrence of anomalous *ASIP* genotypes. We document the detection of combinations of three or four *ASIP* allele variants in 17 dog breeds and Dingoes, at within-breed frequencies of 1.32–63.34%. We analyze the potential impact on phenotype that these allele combinations present, and propose mechanisms that could account for the findings, including: gene recombination, duplication, and incorrect causal variant identification. These findings speak to the accuracy of industry-wide protocols for commercial *ASIP* genotyping and imply that *ASIP* should be analyzed via haplotype, rather than using only the existing allele hierarchy, in the future.

## 1. Introduction

Canine coat color genetics is a subject of much interest to members of the dog breeding community, and the inherently visible and predominantly non-deleterious phenotypic presentation has encouraged many genetic research endeavors. Numerous commercial genotyping laboratories offer basic coat color assays. A recent publication documenting comprehensive color allele frequencies across breeds made note of an unexpected combination of alleles at the *Agouti Signaling Protein* (*ASIP*) gene [1]. The conventional understanding and nomenclature of *ASIP* inheritance consists of four characterized alleles, present in a dominance hierarchy: *a^y^* > *a^w^* > *a^t^* > *a*. The *a^y^* allele is the most dominant and is responsible for fawn coloring, which consists of pheomelanin (yellow- or red-based pigment) at the base of a hair with a eumelanin (black- or brown-based pigment) at the tip [2]. On a molecular level, this allele is characterized by two neighboring amino acid substitutions, A82S and R83H, in exon 4 of *ASIP* [2]. The *a^w^* allele, which is the wild-type allele of *ASIP*, produces a wolf-sable banded hair pattern. When genotyping an individual, the *a^w^* allele is typically identified through a process of elimination when indicators for all other currently-tested *ASIP* alleles are absent [3]. This means that the *a^w^* “allele” is actually a haplotype representing the absence of any of the variant alleles at the *a^y^, a^t^,* or *a* loci. The *a^t^* allele is responsible for producing the tan points phenotype, with eumelanin on the torso of the dog and phaeomelanin on the face and extremities, and is due to a reverse SINE insertion at chr24:23,365,298–23,365,537 in intron 1 of *ASIP* [3]. The most recessive allele, *a*, produces a solid eumelanin color. The *a* allele is characterized by an R96C amino acid substitution in exon 4 of *ASIP* and occurs in conjunction with the SINE insertion found in *a^t^*, thus the *a* allele has both the R96C mutation in exon 4 and the intron 1 SINE insertion [3,4]; these two variants are typically considered to be linked. The current practice of most commercial genetic testing laboratories offering coat color results is to do the following for *ASIP* for each dog: (1) test each variant locus (*a^y^, a^t^,* and *a*) in *ASIP*, (2) identify which variant alleles are present at these three loci, (3) call *a^w^* as present in one copy when only one variant allele is detected at the three tested loci, or call *a^w^* as present in two copies when no variant alleles are detected, and (4) report a forced biallelic genotype, which is then generally interpreted according to the dominance hierarchy outlined above, with the most dominant allele present assumed to be dictating the phenotype. The recent publication of commercial laboratory-determined color allele frequencies, for which numerous authors overlap with those on this manuscript, noted the occurrence of what appeared to be three *ASIP* allele variants in multiple dogs [1]. At the time, this was interpreted to be the existence of the *a^y^* mutation and the *a^t^* mutation occurring on the same chromosome, and was termed the *a^yt^* allele [1]. Subsequent genotype and phenotype data collection now allows us to present a more complete characterization of allele discrepancies at the *ASIP* locus in dogs, propose mechanisms for their occurrence, and outline the implications of such a finding. For the purpose of entertaining multiple possible explanations for the occurrence of these genotyping anomalies, we will herein refer to dogs genotyped with any combination of three or more currently-tested *ASIP* allele variants (*a^y^*, *a^t^*, or *a*) as *A*_3+_. Using data generated via commercial laboratory coat color genotype-testing, with direct testing of published variants, our goal was to describe the existence of *A*_3+_ alleles, report which breeds they are observed in and the frequencies of these results by breed, and present observed phenotypes for *A*_3+_ dogs. Nomenclature as it currently appears in the published canine *ASIP* literature is used, and we suggest both future work and a shift in describing and analyzing the canine *ASIP* gene.

## 2. Materials and Methods

### 2.1. Datasets

Three datasets were independently considered for analysis (Table 1). The results of a prior study conducted by some of the current authors were utilized as published [1]; this study presented data as collected by Wisdom Health for their WISDOM PANEL analysis (Wisdom Health, Vancouver, WA, USA). In this initial study, referred to herein as Wisdom Health: True Colors (WHTC), the occurrence of unexpected combinations of *ASIP* allele variants (*A*_3+_) was observed in 679 dogs (out of 11,790) representing 14 breeds and the Dingo. This cohort has been well-described previously [1]. A new cohort consisting of 3189 purebred dogs representing 14 of these same breeds, plus three additional breeds not included in the initial study, was compiled and will be hence referred to as Wisdom Health: Agouti Expansion (WHAE). This cohort was selected based on breeds identified with the *a^yt^* allele from [1] and during routine data quality control at Wisdom Health. Some overlap in dogs does occur between WHTC and WHAE. The WHAE cohort was collected by Wisdom Health, Helsinki, Finland (formerly Genoscoper Laboratories) between July 2013 and September 2019. Most of the dogs in the WHAE cohort were from Finland (~one third) and the USA (~one quarter) with the remaining dogs representing a roughly global population. All Wisdom Health-generated data was collected and genotyped using the same protocols, as outlined below.

Other commercial laboratories provide canine *ASIP* allele genotyping, therefore, a supplemental cohort consisting of owner-reported *ASIP* genotypes, as produced by any one of six alternate (non-Wisdom Health) commercial laboratories, was assembled. This cohort contains 119 dogs representing three breeds and will be referred to as the Multi-Laboratory Agouti Supplement (MLAS). The supplemental cohort was genotyped using the proprietary protocols consistent with their respective commercial laboratories, and were reported as a biallelic genotype. Each of these laboratories has reported the occurrence of *A*_3+_ as an *a^yt^* allele in a forced biallelic genotype scenario.

Phenotype data (coat color) was available for only a small number of dogs (*n* = 54) from the WHAE and MLAS cohorts.

### 2.2. Sample Collection and Genotyping

For the WHTC and WHAE cohorts, all genetic analyses were carried out on DNA extracted from owner-collected, non-invasive buccal swab samples, or from blood/cheek swab samples collected at certified veterinary clinics in accordance with international standards for animal care and research. All dogs were owned as pets, and samples were voluntarily submitted by owners for commercial screening. All dog owners provided consent for use of their dog’s DNA for research purposes. Therefore, no additional Institutional Animal Care and Use Committee oversight is required. Where applicable, drawing of blood samples was approved by the Animal Ethics Committee of the State Provincial Office of Southern Finland, Hämeenlinna, Finland (permit number: ESAVI/6054/04.10.03/2012). Dogs were defined as purebred for the purposes of this study if registered with: Federation Cynologique Internationale, American Kennel Club, United Kennel Club, the UK Kennel Club, or an applicable single-breed registry for rare breeds.

DNA was extracted using standard protocols, and all dogs were analyzed on the WISDOM PANEL, MYDOGDNA, or OPTIMAL SELECTION platforms (Wisdom Health). Genotyping of all *ASIP* variants (*a^y^*, *a^t^*, and *a*) was conducted on a custom-designed low-density Illumina Infinium bead chip using manufacturer-recommended protocols (Illumina, San Diego, CA, USA); the validation and genotyping quality control measures for this platform were previously described in detail [5,6]. A sample inclusion criteria call rate of 98% for all analyzed markers was enforced, and all genotype calls were manually curated. Bi-allelic genotypes for *ASIP* are obtained by testing for the occurrence of the *a^y^* A82S and/or R83H mutation (depending on the commercial laboratory; for the present study, all WHTC and WHAE dogs were genotyped for both alleles, and the alleles are confirmed to be in full linkage for all dogs in the WHAE cohort), the *a* R96C mutation, and the SINE insertion attributed to the *a^t^* and *a* alleles. The absence of these mutations indicates the presence of the wild-type *a^w^* allele. For the purpose of this study, the raw genotypes at each of the mutations for all dogs in the WHTC and WHAE datasets were annotated separately and not transformed into the bi-allelic nomenclature. Genotypes were also obtained for the *Melanocortin 1 Receptor* (*MC1R*) [7,8] and *Canine β-Defensin 103* (*CBD103*) [9,10] for phenotype analysis, as certain genotypes at these loci will mask expression of *ASIP*.

The individual *a^y^* and *a^t^* allele genotypes detected as tri-allelic via the bead chip were confirmed in two dogs by standard capillary sequencing on an ABI3730 × 1 DNA Analyzer platform (ThermoFisher Scientific, Waltham, MA, USA) at the Sequencing Unit of the Finnish Institute of Molecular Medicine (Figure S1). Preparation and purification of PCR products for sequencing was carried out following manufacturer’s instructions, as previously described in detail [5,6]. Primer sequences are provided in Table S2, and all primers were verified to work on control (non-*A*_3+_) dogs.

## 3. Results

### 3.1. Detected Frequencies of A_3+_

The WHAE cohort consists of 3189 dogs, of which 158 dogs, representing 12 of the 17 breeds, were found to be *A*_3+_ (Table 1), a frequency of 4.95%. The proportion of individual dogs in each breed that genotyped as *A*_3+_ ranges from 1.32% (Whippet) to 63.64% (Dogo Argentino). The WHTC dataset had an *A*_3+_ frequency of 6.63% across 14 breeds plus Dingoes [1]. Five breeds (Berger Picard, Boston Terrier, Brussels Griffon, Great Dane, and Irish Terrier), identified in WHTC as consisting of at least one *A*_3+_ dog, were included in the WHAE dataset, and no additional incidents of *A*_3+_ dogs were identified. Conversely, three breeds (Basenji, East-Siberian Laika, and Kai Ken), in which *A*_3+_ was newly observed, were included in the WHAE dataset, but were not included in the WHTC dataset. Owner-reported genotypes obtained from any one of six other commercial testing laboratories (unaffiliated with the authors of this study) were recorded for 119 dogs (MLAS). Nineteen of these dogs were genotyped as *A*_3+_ (reported as an *a^yt^* allele), supporting that the detection of these anomalies is not dependent on laboratory-specific protocols, and is therefore not a simple genotyping error.

Observed allele combinations in WHAE consist of all potential *ASIP* bi-allelic pairs, with the exception of *a^w^*/*a* (Table 2). Three different combinations of three alleles were observed, and a single combination of four alleles was observed in seven separate dogs.

### 3.2. Phenotypic Impact of A_3+_

**Figure 1:**
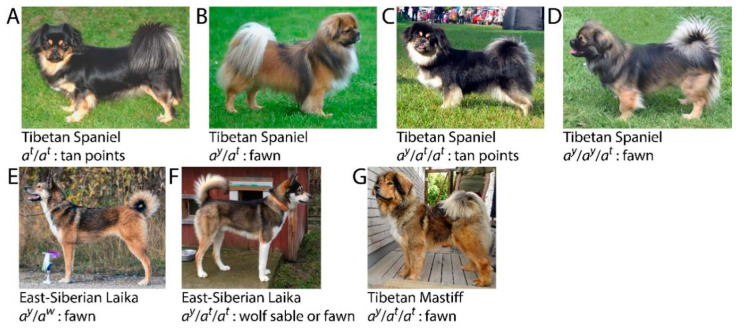
Conventional and *A_3+_ ASIP* genotype-phenotype combinations. (**A**–**D**) Tibetan Spaniels routinely express a tan point phenotype (**A**), produced by homozygous *a^t^* alleles, and a fawn phenotype (**B**), produced with a dominant *a^y^* allele. When genotyped as *A*_3+_ (**C**,**D**), the allele that is present twice will dictate the phenotype. East-Siberian Laikas (**E**,**F**) possess all four known *ASIP* alleles naturally. The dominant *a^y^* allele produces a fawn phenotype (**E**), while an East-Siberian Laika genotyped as *a^y^*/*a^t^*/*a^t^* (**F**) could be *a^y^* fawn or *a^w^* wolf sable. In Tibetan Mastiffs (**G**) with *a^y^/a^t^/a^t^*, a fawn phenotype is expressed, a different pattern than that seen in Tibetan Spaniels (**C**).

Color phenotypes were available for 54 *A*_3+_ dogs from the WHAE and MLAS cohorts, after the removal of dogs that were solid eumelanin due to the *K^B^* allele of *CBD103* [9,10], solid phaeomelanin due to a homozygous *e*/*e* genotype at *MC1R* [8], or had white spotting patterns that effectively obscured the expressed *ASIP* phenotype. Four *A*_3+_ combinations were observed (Table 3), and some phenotypes were different than what would be expected under the existing allele hierarchy as described in the literature. If allele combinations and phenotypes are considered within breeds, multiple expression patterns begin to emerge. Thirty-two Tibetan Spaniels with known phenotypes have three allele variants each. Within this breed, the phenotype expressed is the one resultant of the allele that is present twice (Figure 1). For instance, a genotype of *a^y^*/*a^y^*/*a^t^* produces the *a^y^* fawn phenotype, while a genotype of *a^y^*/*a^t^*/*a^t^* produces the *a^t^* tan points phenotype. However, this pattern does not reflect the expression of alleles in other breeds, such as the Tibetan Mastiff. Four Tibetan Mastiffs with known phenotypes have been genotyped with three alleles, and in this breed both the *a^y^*/*a^y^*/*a^t^* and *a^y^*/*a^t^*/*a^t^* combinations produce an *a^y^* fawn phenotype (Figure 1). Eleven East-Siberian Laikas were genotyped as having three or four *ASIP* alleles and also have available phenotypic information (Table 3). The allele combinations of *a^y^*/*a^t^*/*a*, and *a^y^*/*a^y^*/*a^t^*/*a^t^* can produce phenotypes consistent with the *a^w^* wild-type allele (wolf sable), while the single East-Siberian Laika genotyped as *a^y^*/*a^y^*/*a^t^* shows a fawn phenotype. In East-Siberian Laikas and across different breed backgrounds, the *a^y^*/*a^t^*/*a^t^* combination produced variable phenotypes, including the *a^w^* wild-type (wolf sable), *a^y^* fawn, and *a^t^* tan points (Figure 1).

## 4. Discussion

**Figure 2:**
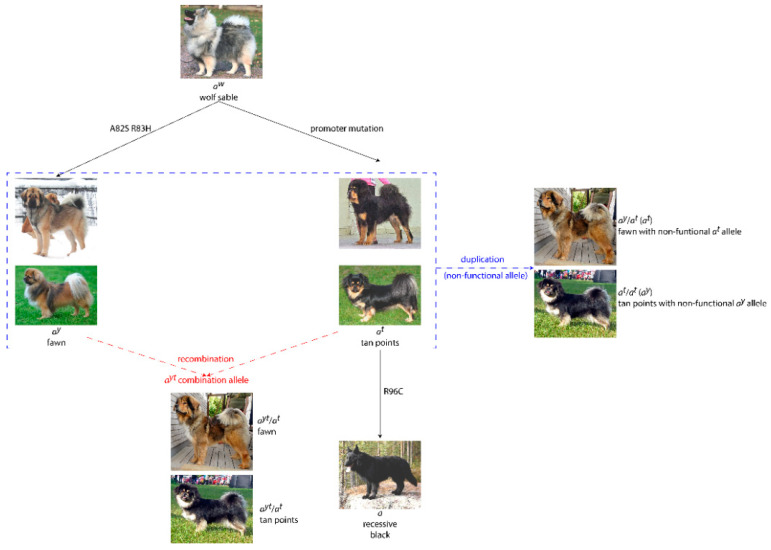
The evolution of *ASIP* alleles with alternate means by which *A*_3+_ may have arisen. Blue dashed lines outline the possible duplication of the *a^y^* and *a^t^* alleles into non-functional copies. Red dashed lines indicate the recombination of the *a^y^* and *a^t^* alleles into a combined *a^yt^* allele. An example of a fawn colored Tibetan Mastiff and a tan-pointed Tibetan Spaniel, both having been genotyped as *a^y^*/*a^t^*/*a^t^*, are used to illustrate each mechanism.

The observation of more than two *ASIP* variants in a single dog presents an intriguing challenge within the small subset of breeds where it has been observed; these breeds will undoubtedly provide the key to ultimately determining what actual chromosomal rearrangement(s) have occurred. The breeds in which this scenario is observed range from molossoid flock guardians, to sight hounds, to companion breeds. Previous research suggests potential relatedness between some of these breeds, such as the Tibetan Spaniel and Tibetan Mastiff or the Maremma Sheepdog and Great Pyrenees, though these relationships point to distant common ancestral origins of multiple breeds rather than direct breed-to-breed progression [11,12]. Potential causes of the *A*_3+_ anomalies could be attributed to at least three different scenarios: (1) recombination within the *ASIP* gene, (2) duplication of part or all of the *ASIP* gene, or (3) an accepted *ASIP* allele is incorrectly attributed to and tested via an incompletely linked marker variant (Figure 2). When considering recombination as a potential explanation for the seeming appearance of multiple allele variants on the same chromosome, the positioning of the annotated allele variants must be considered. The SINE insertion in intron 1 is found in dogs with the tan point phenotype and, in conjunction with an R96C amino acid substitution in exon 4, in dogs with the recessive black phenotype. The *a^y^* allele, attributed to two adjacent amino acid substitutions at A82S R83H, is also in exon 4, thirteen codons upstream of the *a* allele variant. The genomic distance between the *a^t^* SINE and *ASIP* exon 4 is approximately 27 kb. With an estimated recombination frequency of 1% per 1 cM, and a canine correction estimate of 1.55 cM/Mb [13], the frequency of recombination between the *a^t^* SINE and exon 4 is 0.042%. We have observed dogs with *A*_3+_ genotypes involving the *a* allele, though these situations are such that they may consist of a normal *a* allele and a rearrangement only incorporating the other two alleles. Therefore, a recombination between the *a* and *a^y^* variants, situated at a distance of 39 bp apart, is not required for *A*_3+_ that includes an *a* allele. Under a recombination model, different breeds could display variable phenotype expression patterns of the *A*_3+_ allele variants.

An alternative theory to explain the detection of *A*_3+_ allele variants involves the partial or complete duplication of *ASIP* in a small number of dog breeds. The duplication, if non-functional, would allow for the detection of an allele variant without an observable impact on the expressed phenotype. In this way, assays designed to amplify genomic regions immediately surrounding the known variants would inadvertently identify the functional and non-functional gene copies. The duplication would be variable in terms of allele variants present, and also variable in the frequency of its occurrence, with the majority of dogs not possessing the duplication and genotyping as bi-allelic. Using the Tibetan Spaniel as an example (Figure 1), a dog with a tan point phenotype with a genotype of *a^y^*/*a^t^*/*a^t^* would have a functional *ASIP* genotype of *a^t^*/*a^t^* and a single duplication copy with the *a^y^* variant. Conversely, a fawn Tibetan Spaniel with a genotype of *a^y^*/*a^y^*/*a^t^* would have a functional *ASIP* genotype of either *a^y^*/*a^t^* or *a^y^*/*a^y^*, and the single duplication could be either *a^y^* or *a^t^*, respectively. Some breeds that are observed to have the *a^y^*/*a^y^*/*a^t^* combination are also observed to have a very low frequency of the *a^t^* allele among dogs with a bi-allelic genotype. An example of this is the Whippet, which has a bi-allelic genotype allele frequency of 0.83% for the *a^t^* allele. The three Whippets identified in this study that have an allele combination of *a^y^*/*a^y^*/*a^t^* present a fawn phenotype (with brindle and/or white markings), consistent with the *a^y^* allele that is predominant in the breed. That would suggest, therefore, that the duplicated non-functional allele in this breed is the *a^t^*.

Both the duplication and recombination theories could explain the variable inheritance patterns in different breeds and even the expression of the *a^w^* phenotype in the absence of a detectable *a^w^* allele. Under a recombination model with *a^yt^* on one chromosome, where the Illumina SNP genotyping methodology would indicate heterozygosity at both the *a^y^* and *a^t^* loci, an *a^w^* haplotype could be present and not accurately detectable. If all alleles were functional, the dog should have a fawn (*a^y^*-dictated) phenotype, but would have an undetected *a^w^* chromosome that could be passed to offspring. Or, if the *a^y^* were non-functional, a dog could test as *a^y^/a^t^* and still have an *a^w^* phenotype. Such scenarios must be considered in breeds with a known occurrence of the *a^w^* allele, such as the East-Siberian Laika. Conversely, a homozygous result at a variant locus (such as *a^t^/a^t^*) means that no wild-type allele is present at that locus when using the Illumina SNP genotyping methodology, and this indicates that the normal *a^w^* haplotype is not present. Intermediate coat color phenotypes could still be observed due to accumulation of *ASIP* mutations, including combinations of gain- or loss-of-function alleles, under a duplication model. *A*_3+_ scenarios are more difficult to elucidate phenotypically: in Figure 1F, the East-Siberian Laika presented has genotyped as *a^y^*/*a^t^*/*a^t^*, and presents a phenotype that could be consistent with a darkened form of *a^y^* fawn or the *a^w^* allele (haplotype), despite the fact that the homozygous *a^t^/a^t^* result means there is no wild-type allele at that locus (*a^w^* is not present). This ambiguity would allow for a possible intermediate phenotype of appearing more wolf sable (*a^w^*) due to the combination of accruing variant alleles, or appearing as a darkened fawn (*a^y^*/*a^t^*), with the additional *a^t^* allele present as a non-functional duplication.

The suggested duplication theory would require that a duplication event would have occurred multiple times in order for both the *a^y^* and *a^t^* allele variants to be variably represented. A similar scenario of gene duplication with variable alleles has been observed in dogs before, specifically in regard to the production of the brindle phenotype. The brindle phenotype was shown to represent an allele of the *CBD103* gene, *k^br^*, dominant to *k^y^* and recessive to *K^B^* [9]. The molecular nature of *k^br^* is a structural variant that affects gene copy number (personal communication, Drs. Barsh and Kaelin). Copy number variation of the *ASIP* gene has been reported to occur, and impact coat color, in domestic goats and sheep [14,15,16,17,18,19]. For these species, the copy number variant is purported to be functional, impacting pigmentation expression. A functional *ASIP* duplication in dogs, though as yet unsubstantiated, may also affect pigmentation and shading variation present in dogs with fawn, tan point, and wild-type (wolf sable) phenotypes. If any duplications have occurred, this increase in allele number for each of the tested variants could potentially create genotyping and/or test result interpretation errors. In reality, a recombination between the *a^y^* and *a^t^* variants is not less likely than a scenario with multiple duplications. Mechanistic evaluations will be required to tease out these scenarios, always with the consideration that other coat color genes, both known and any yet undescribed, can influence the phenotype.

A third potential cause for the *A*_3+_ anomalies is the possibility that one or more of the identified allele variants of *ASIP* is actually a highly-linked marker and not a causal variant. We did not have phenotypes or family pedigree structure for most of the dogs in the present study, therefore we could not conduct association or linkage analyses in further investigations. While the exonic variants associated with *a^y^* and *a*, and the intronic SINE insertion attributed to *a^t^* have been widely used commercially to predict inheritance and expression of coat colors and patterns, genotype discrepancies such as those detailed here could be caused by incomplete linkage between the assayed and causal variants. Alternate causal variants for the tan point phenotype have been proposed [20], though are not broadly incorporated into commercial testing panels.

These data highlight the necessity for additional investigation into the occurrence of multiple allele variants in relation to recombination events or the occurrence of *ASIP* gene duplications. For example, investigations could utilize DNA from dogs with known atypical genotypes in droplet digital PCR, long-range sequencing, and/or on commercially-available highly dense SNP arrays where SNP data can be accurately phased into haplotypes and used in linkage disequilibrium analyses and/or where raw SNP data intensity values can be analyzed for copy number changes. Accurate phenotypes and pedigrees would also facilitate teasing out haplotype-to-phenotype associations and linkage/recombination, not only on dogs with atypical *ASIP* results, but also across all dogs for verification. Any haplotype-to-phenotype analyses must, of course, keep other genes with epistatic functions in mind (e.g., *MC1R, CBD103*). Future work should also explore *ASIP* expression, which may help further decipher genotype/haplotype-to-phenotype correlations. The East-Siberian Laika, in particular, ought to have its *ASIP* gene sequence examined in its entirety, due to the incongruous genotype–phenotype observations.

With four already-published variants, including the wild type *a^w^* haplotype, in the canine *ASIP* gene, it is clear that this area of the genome is prone to change, and also clear that these changes are actively selected for in different breeds of domestic dog. Multiple additional species report point mutations, small insertions/deletions, and copy number variants of *ASIP* as causal for coat color phenotypes, suggesting great plasticity of this gene across species [18,19,21,22,23,24]. Therefore, it is reasonable to consider that either recombination or duplication, or both, could have occurred at this locus in dogs, possibly multiple times. It is probably wise to also consider that: (1) other alleles might exist at the known variants, (2) other variants might exist within the canine *ASIP* gene, and (3) linkage across the canine *ASIP* gene may not be 100%.

In addition, epigenetic mechanisms, such as methylation, could be in play. Variable coat color expression is seen in mice with a retrotransposon upstream of the *agouti* gene [25], where the range of color inversely correlates with DNA methylation states [26,27]. Such metastable epialleles, with variable phenotypes correlating directly with an epigenetic state, are not uncommon in mammalian genomes [28], making this yet another potential mechanism that must be considered in future investigations of canine *ASIP* expression.

Lastly, we have identified a situation that poses a challenge to commercial laboratories offering coat color genotyping, whereby a small portion of dogs will not correctly genotype for *ASIP* if the *a^w^* allele is masked by a recombination situation. The current data identifies six breeds for which this scenario definitely exists, given the determination of an *a^w^* allele through exclusion of *a^t^*, *a^y^*, and *a* variants: Basenji, East-Siberian Laika, Great Pyrenees, Kai Ken, Lagotto Romagnolo, and Maremma Sheepdog. Under the same exclusion-based genotyping of *a^w^*, the previously published WHTC dataset adds the Anatolian Shepherd, Great Dane, Tibetan Mastiff, and Tibetan Spaniel breeds to this list, for a minimum of ten breeds that have some positive frequency of the *a^w^* allele and at least one observed incidence of greater than two identified *ASIP* variant alleles in a single dog [1]. Commercial laboratories should consider presenting a disclaimer with reported *ASIP* genotypes, particularly in breeds known to possess the *a^w^* allele (haplotype), that the reported presence or absence of that haplotype cannot be accurately detected in certain cases. In instances where a dog appears to present an *a^w^* phenotype in the absence of the appropriate genotype, it should be considered whether the breed in question may be affected in this context.

The current approach to understanding and interpreting the canine *ASIP* gene has worked well historically and is still valid in many breeds, but the data presented here indicate that it is not always accurate in several breeds. Adjustments are required, including changes in the typically-utilized terminology. Moving forward, it would be best to cease calling *a^w^* the wild-type “allele” and rather call it a haplotype, since it is actually the absence of the variant allele at any of the currently-tested loci on one chromosome. Canine coat color phenotypes, at least in known *A*_3+_ breeds, should be re-assessed via association with *ASIP* haplotypes, rather than defaulting to the existing allele hierarchy methodology. This is due to the evident segregation that is occurring between the alleles in these dogs, which is potentially causing a difference in phenotype compared to the existing literature and prevailing understanding in the dog genetic testing industry. Although *A*_3+_ dogs are rare, we were able to obtain phenotypes for 54 of 158 *A*_3+_ dogs in the present study, allowing us to speculate on the phenotype characterization. With this definitive report of *A*_3+_ dogs, laboratories providing coat color testing results should consider modifying their interpretation and reporting procedures.

## 5. Conclusions

We postulate that these unexpected *A*_3+_ genotypes have not been previously identified due primarily to their very low frequency across purebred dogs as a whole. The previous report indicated that greater than two *ASIP* alleles occurred in a single dog at a frequency of 0.41%, out of 11,790 dogs [1]. The recent increased accessibility and popularity of commercially-available canine genotyping panels has allowed for the data accumulation from sample sizes not previously seen in genetic research of this species, revealing these rare, though biologically relevant, atypical results. Significant additional experimental investigation and shifts in both nomenclature and analysis of the *ASIP* gene are required.

## Figures and Tables

**Table 1:** Dogs from three datasets that genotype for three or more *Agouti Signaling Protein* (*ASIP*) alleles (*A*_3+_). The proportion of same-breed dogs that genotype as *A*_3+_ are presented as a percentage of the total number of dogs for that breed within the given dataset. Supplemental dogs (Multi-Laboratory Agouti Supplement (MLAS)) were genotyped at any one of six alternate commercial genotyping laboratories. Since these multi-platform dogs were not randomly selected, population percentages were not produced. *ASIP* allele frequencies across cohort, as calculated according to current methodologies in the field, are presented in Table S1.

Breed	WHTC			WHAE			MLAS	
	N	*A*_3+_	%	*n*	*A*_3+_	%	*n*	*A*_3+_
Anatolian Shepherd	22	1	4.55%	18	1	5.56%	-	-
Basenji	-	-	-	74	1	1.35%	-	-
Berger Picard	9	1	11.11%	7	0	0.00%	-	-
Boston Terrier	64	4	6.25%	94	0	0.00%	3	3
Brussels Griffon	44	1	2.27%	44	0	0.00%	-	-
Dingo	12	2	16.67%	-	-	-	-	-
Dogo Argentino	9	6	66.67%	11	7	63.64%	-	-
East-Siberian Laika	-	-	-	109	15	13.76%	-	-
Great Dane	79	1	1.27%	163	0	0.00%	-	-
Great Pyrenees	51	8	15.69%	70	9	12.86%	-	-
Irish Terrier	33	1	3.03%	55	0	0.00%	-	-
Kai Ken	-	-	-	19	1	5.26%	-	-
Lagotto Romagnolo	116	8	6.90%	1840	80	4.35%	-	-
Maremma Sheepdog	18	3	16.67%	24	5	20.83%	-	-
Spanish Greyhound	25	1	4.00%	72	1	1.39%	-	-
Tibetan Mastiff	83	6	7.23%	108	14	12.96%	1	1
Tibetan Spaniel	46	1	2.17%	177	20	11.30%	115	15
Whippet	68	1	1.47%	304	4	1.32%	-	-
Total	679	45		3189	158		119	19

**Table 2:** Detected allele combinations in the Wisdom Health: Agouti Expansion (WHAE) cohort of 3189 dogs representing 17 breeds. The current dataset includes 158 dogs with *A*_3+_ genotypes. Genotypes obtained for three *ASIP* mutations have traditionally been used to produce bi-allelic genotypes with a four allele hierarchy. Raw genotypes for *a^y^*, *a^t^*, and *a* are encoded as 1 = reference, 2 = alternate.

No. of Non-Wild-Type Variants ^a^	*a^y^* [2]	*a^t^* [3]	*a* [4]	Interpreted Genotype ^b^	No. of Observations (Out of 3189 Dogs)	Proportion of Genotypes
	p.A82S	g.23365298ins239	p.R96C			
2	2/2	1/1	1/1	*a^y^*/*a^y^*	909	28.50%
1	1/2	1/1	1/1	*a^y^*/*a^w^*	29	0.91%
2	1/2	1/2	1/1	*a^y^*/*a^t^*	376	11.79%
2	1/2	1/2	1/2	*a^y^*/*a*	44	1.38%
0	1/1	1/1	1/1	*a^w^*/*a^w^*	13	0.41%
1	1/1	1/2	1/1	*a^w^*/*a^t^*	62	1.94%
1	1/1	1/2	1/2	*a^w^*/*a*	0	0.00%
2	1/1	2/2	1/1	*a^t^*/*a^t^*	1303	40.86%
2	1/1	2/2	1/2	*a^t^*/*a*	265	8.31%
2	1/1	2/2	2/2	*a*/*a*	30	0.94%
3	2/2	1/2	1/1	*a^y^*/*a^y^*/*a^t^*	41	1.29%
3	1/2	2/2	1/1	*a^y^*/*a^t^*/*a^t^*	92	2.88%
3	1/2	2/2	1/2	*a^y^*/*a^t^*/*a*	18	0.56%
4	2/2	2/2	1/1	*a^y^*/*a^y^*/*a^t^*/*a^t^*	7	0.22%

^a^ In the currently-utilized commercial genetic testing methodology, which determines the alleles present at the three *ASIP* variant loci, under the assumption that (1) *a^w^* is present in one or two copies when one or zero variant alleles are present, respectively, and (2) where *a*, when present, is in linkage with *a^t^*. ^b^ Genotypes as they would be interpreted according to the current literature’s published hierarchy, and according to typical current commercial testing laboratory protocols.

**Table 3:** Phenotypes expressed with each observed *ASIP* allele combination. Phenotypes are available for some dogs (total *n* = 54) of the WHAE and MLAS cohorts. The “phenotype allele” refers to the allele that would be expected to produce the observed phenotype.

Alleles	Breed	*n*	Color	Phenotype Allele
*a^y^*/*a^y^*/*a^t^*	Boston Terrier	3	fawn (with brindle)	*a^y^*
	East-Siberian Laika	1	fawn	*a^y^*
	Great Pyrenees	1	fawn (with white)	*a^y^*
	Tibetan Mastiff	1	fawn	*a^y^*
	Tibetan Spaniel	20	fawn	*a^y^*
	Whippet	3	fawn (with brindle)	*a^y^*
*a^y^*/*a^t^*/*a^t^*	East-Siberian Laika	1	wolf sable	*a^w^*
		2	dark fawn or wolf sable	*a^y^* or *a^w^*
		2	fawn	*a^y^*
		1	tan points	*a^t^*
	Tibetan Mastiff	3	fawn	*a^y^*
	Tibetan Spaniel	12	tan points	*a^t^*
*a^y^*/*a^t^*/*a*	East-Siberian Laika	2	wolf sable	*a^w^*
*a^y^*/*a^y^*/*a^t^*/*a^t^*	East-Siberian Laika	1	wolf sable	*a^w^*
	Whippet	1	fawn (with brindle)	*a^y^*

## Supplementary Materials

The following are available online at https://www.mdpi.com/2073-4425/11/7/739/s1, Table S1: Overall frequencies of *ASIP* variants in each cohort. Frequencies calculated for WHTC and WHAE cohorts are based on the assumption that each dog has two alleles; this is likely not the case in reality, therefore these numbers are presumed to be slightly inaccurate. MLAS frequencies not provided because the sample recruitment was not random. Table S2: Primers used in verification Sanger sequencing. Table S3: Complete data *ASIP* genotypes for WHAE and MLAS cohorts. A1 = ancestral (wild-type) allele, A2 = derived (variant) allele. Figure S1: Sanger sequencing chromatograms of two *A*_3+_ dogs. Sanger sequencing was performed to confirm the Illumina SNP genotypes, and to verify that no nearby variants would have resulted in missing detection of an allele. Both dogs are *A*_3+_ Tibetan Spaniels, specifically, both dogs are homozygous for the *a^t^* insertion and heterozygous for both *a^y^* missense variants, resulting in an interpreted genotype of *a^y^*/*a^t^*/*a^t^*. These were the only two samples from *A*_3+_ dogs with DNA available for additional sequencing. Primers (Table S2) were verified to work on control (non-*A^3+^*) samples.
