# Supplementary material for: Atypical Genotypes for Canine Agouti Signaling Protein Suggest Novel Chromosomal Rearrangement
Source: Genes (Basel). 2020 Jul 3;11(7):739. doi: 10.3390/genes11070739 (PMC7397341; doi:10.3390/genes11070739)

**Figure S1: Sanger sequencing chromatograms of two *A_3+_* dogs.** Sanger sequencing was performed to confirm the Illumina SNP genotypes, and to verify that no nearby variants would have resulted in missing detection of an allele. Both dogs are *A_3+_* Tibetan Spaniels, specifically, both dogs are homozygous for the *a^t^* insertion and heterozygous for both *a^y^* missense variants, resulting in an interpreted genotype of *a^y^*/*a^t^*/*a^t^*. These were the only two samples from *A_3+_* dogs with DNA available for additional sequencing. Primers (Table S2) were verified to work on control (non-*A^3+^*) samples.

First sequenced Tibetan Spaniel:

Homozygous SINE insertion. Interpreted as two *a^t^* alleles.


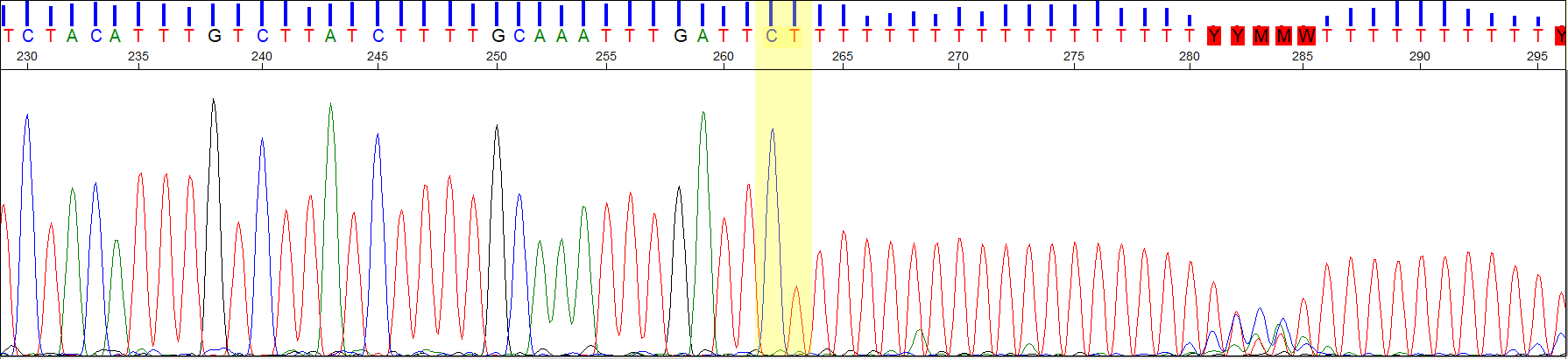


Heterozygous for two *a^y^* missense variants, shown here as Y and M (within yellow highlighted region).

Interpreted as one *a^y^* allele.


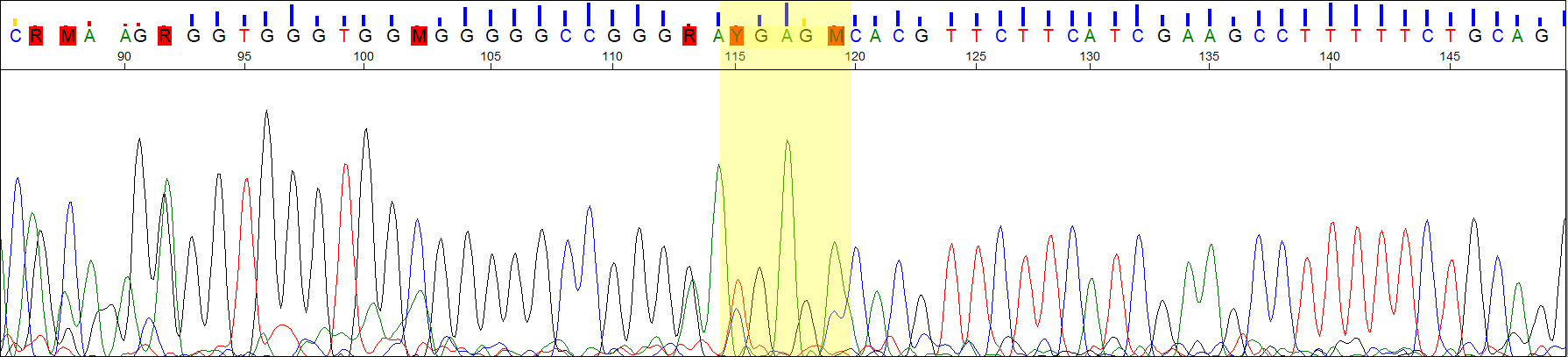


Second sequenced Tibetan Spaniel:

Homozygous SINE insertion (interpreted as two *a^t^* alleles)


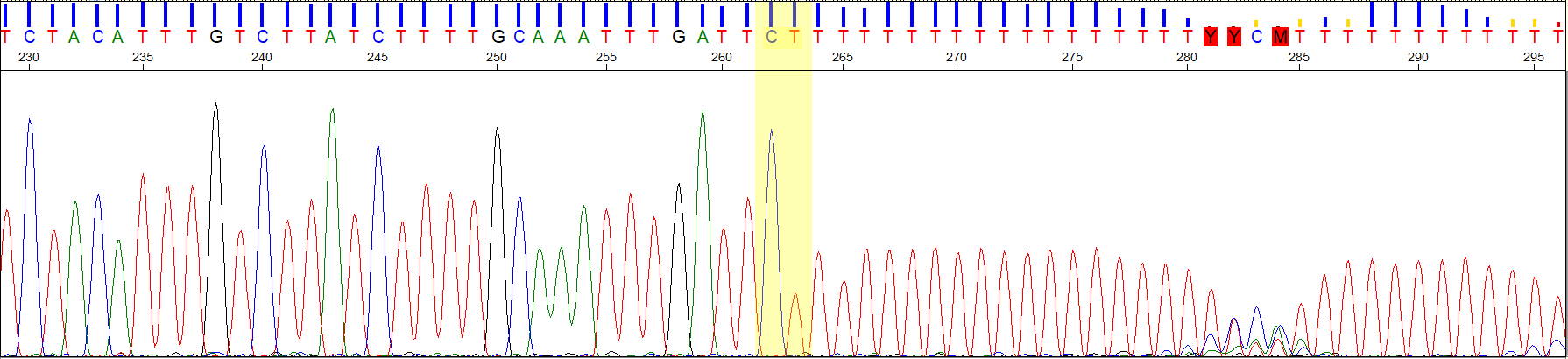


Heterozygous for two *a^y^* missense variants, shown here as Y and M (within yellow highlighted region).

Interpreted as one *a^y^* allele.


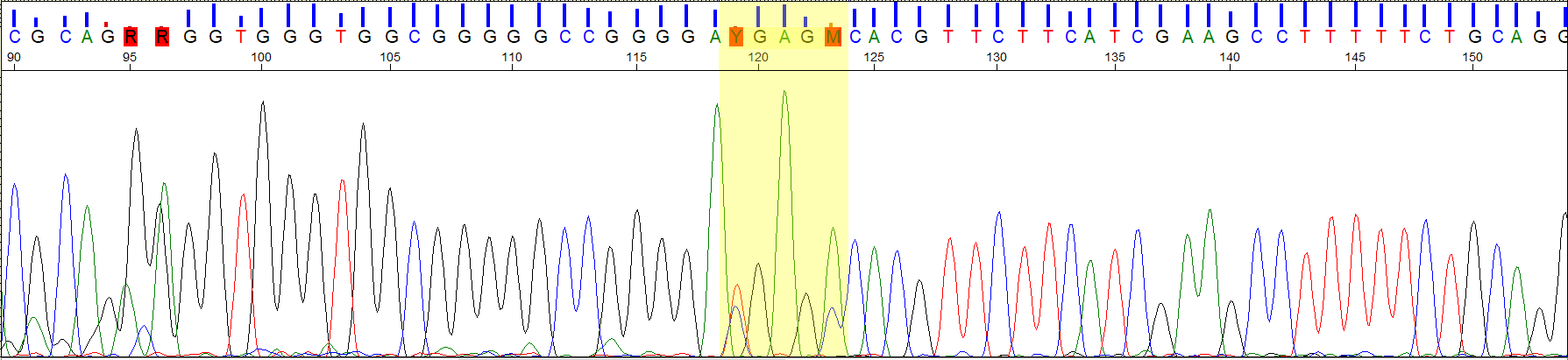

Supplement: Supplementary file 1 [file genes-11-00739-s001.zip › Supplemental Materials/Figure S1.docx]
